# Impaired aldehyde dehydrogenase 1 subfamily member 2A-dependent retinoic acid signaling is related with a mesenchymal-like phenotype and an unfavorable prognosis of head and neck squamous cell carcinoma

**DOI:** 10.1186/s12943-015-0476-0

**Published:** 2015-12-03

**Authors:** Katharina Seidensaal, Andre Nollert, Agnes Hiou Feige, Marie Muller, Thomas Fleming, Nikolas Gunkel, Karim Zaoui, Niels Grabe, Wilko Weichert, Klaus-Josef Weber, Peter Plinkert, Christian Simon, Jochen Hess

**Affiliations:** Department of Otolaryngology, Head and Neck Surgery, Section Experimental and Translational Head and Neck Oncology, University Hospital Heidelberg, Im Neuenheimer Feld 400, D-69120 Heidelberg, Germany; Research Group Molecular Mechanisms of Head and Neck Tumor, German Cancer Research Center (DKFZ), Heidelberg, Germany; Service d’Oto-Rhino-Laryngologie et Chirurgie Cervico-Faciale, Centre Hospitalier Universitaire Vaudois (CHUV), Université Lausanne, Lausanne, Switzerland; Department of Medicine I and Clinical Chemistry, University Hospital Heidelberg, Heidelberg, Germany; Cancer Drug Discovery, German Cancer Research Center Heidelberg, Heidelberg, Germany; Hamamatsu Tissue Imaging and Analysis Center (TIGA), BIOQUANT, Heidelberg, Germany; Medical Oncology, National Center for Tumor Diseases (NCT), Heidelberg, Germany; Institute of Pathology, University Hospital Heidelberg, Heidelberg, Germany; National Center for Tumor Diseases (NCT), Heidelberg, Germany; Institute of Pathology, Technical University Munich (TUM), Munich, Germany; Department of Radiation Oncology, University Hospital Heidelberg, Heidelberg, Germany

**Keywords:** ALDH1A2, ATRA, BMS493, CRABP2, HNSCC, OPSCC, RAR, Vimentin

## Abstract

**Background:**

An inverse correlation between expression of the aldehyde dehydrogenase 1 subfamily A2 (ALDH1A2) and gene promoter methylation has been identified as a common feature of oropharyngeal squamous cell carcinoma (OPSCC). Moreover, low ALDH1A2 expression was associated with an unfavorable prognosis of OPSCC patients, however the causal link between reduced ALDH1A2 function and treatment failure has not been addressed so far.

**Methods:**

Serial sections from tissue microarrays of patients with primary OPSCC (*n* = 101) were stained by immunohistochemistry for key regulators of retinoic acid (RA) signaling, including ALDH1A2. Survival with respect to these regulators was investigated by univariate Kaplan-Meier analysis and multivariate Cox regression proportional hazard models. The impact of ALDH1A2-RAR signaling on tumor-relevant processes was addressed in established tumor cell lines and in an orthotopic mouse xenograft model.

**Results:**

Immunohistochemical analysis showed an improved prognosis of ALDH1A2^high^ OPSCC only in the presence of CRABP2, an intracellular RA transporter. Moreover, an ALDH1A2^high^CRABP2^high^ staining pattern served as an independent predictor for progression-free (HR: 0.395, *p* = 0.007) and overall survival (HR: 0.303, *p* = 0.002), suggesting a critical impact of RA metabolism and signaling on clinical outcome. Functionally, ALDH1A2 expression and activity in tumor cell lines were related to RA levels. While administration of retinoids inhibited clonogenic growth and proliferation, the pharmacological inhibition of ALDH1A2-RAR signaling resulted in loss of cell-cell adhesion and a mesenchymal-like phenotype. Xenograft tumors derived from FaDu cells with stable silencing of ALDH1A2 and primary tumors from OPSCC patients with low ALDH1A2 expression exhibited a mesenchymal-like phenotype characterized by vimentin expression.

**Conclusions:**

This study has unraveled a critical role of ALDH1A2-RAR signaling in the pathogenesis of head and neck cancer and our data implicate that patients with ALDH1A2^low^ tumors might benefit from adjuvant treatment with retinoids.

**Electronic supplementary material:**

The online version of this article (doi:10.1186/s12943-015-0476-0) contains supplementary material, which is available to authorized users.

## Background

Squamous cell carcinoma of the head and neck (HNSCC) represents one of the most prevalent and lethal human malignancies worldwide, with only a few therapeutic options of limited clinical benefit, available [1]. Consequently, appropriate treatment of advanced HNSCC still remains a major challenge for translational oncology, and there is an urgent need for reliable prognostic biomarkers for treatment failure, as well as innovative drug targets for more effective and less toxic therapy [2]. Functional genomic approaches including global gene expression profiling and whole-exome sequencing have yielded new insights into the molecular principles underlying the pathogenesis and have highlighted the presence of therapeutic candidates in most primary HNSCC [3–5]. However, the causal link and clinical relevance of most differentially expressed genes and somatic mutations for tumor progression and patient survival remains to be addressed considering the extremely high inter- and intratumoral heterogeneity of HNSCC. In addition to genetic events, aberrant DNA hyper-methylation has been linked with the etiology, pathogenesis and clinical outcome of HNSCC patients [6, 7]. However, our knowledge on how distinct genetic and epigenetic alterations affect the tumor response to established or novel therapeutic interventions is still limited.

Previously, we have conducted global DNA methylome analysis on samples of oropharyngeal SCCs (OPSCC), which showed a specific signature of gene promoter methylation that predicted improved clinical outcome in three independent patient cohorts [8]. One candidate gene from our prognosticator encodes for the aldehyde dehydrogenase 1 subfamily member A2 (ALDH1A2), and low ALDH1A2 protein levels served as unfavorable prognostic biomarker for the survival of OPSCC patients [8]. ALDH1A2 is the rate-limiting enzyme in the cellular synthesis of the vitamin A metabolite retinoic acid (RA), which regulates multiple biological processes during embryonic development and in adult tissue remodeling and homeostasis [9]. RA mainly exerts its biological function, including cell differentiation, cell cycle arrest, and eventually apoptosis, through binding to nuclear RA receptors (RAR) [10–12]. Upon ligand binding, RARs form heterodimers with retinoid X receptors (RXR) and function as ligand-dependent transcription factors to activate downstream target genes containing RA response elements (RARE) [11]. In addition to this classical pathway, RARs and RXRs can form heterodimers with other types of nuclear receptors, including the estrogen receptor (ER), peroxisome proliferator-activated receptor (PPAR), liver X receptors (LXR), and vitamin D receptor (VDR) [12]. Interestingly, these non-classical pathways often regulate cellular processes that have functions opposite to the classical pathway.

RA and related synthetic products display potent anticancer activity in certain human malignancies, such as promyelocytic leukemia, which is mainly mediated by activation of the classical RA pathway and forms the basis for its therapeutic application [13, 14]. Although pharmacologic doses of RA derivatives have been effective in the treatment of hematologic malignancies, clinical trials in the prevention and treatment setting of solid tumors, including HNSCC, have failed to show significant benefit [15]. Notably some carcinomas not only fail to become growth inhibited upon treatment with RA, but respond with enhanced proliferation upon treatment. A clue to this paradoxical behavior was recently suggested by the findings that RA also activates PPARβ/δ, a receptor involved in mitogenic and anti-apoptotic activities [16]. In this context, it is worth noting that the distribution of RA between distinct nuclear receptors is regulated by two intracellular lipid-binding proteins, namely cellular RA binding protein 2 (CRABP2), which targets RA to RAR, and fatty acid binding protein 5 (FABP5), which delivers it to PPARβ/δ.

In the current study, we addressed the questions whether improved clinical outcome of ALDH1A2-positive tumors depends on the presence of key regulators of RA signaling. Moreover, we demonstrate that inhibition of ALDH1A2-RAR signaling induces a mesenchymal-like phenotype in vitro as well as in vivo, suggesting that patients with ALDH1A2^low^ tumors might benefit from treatment with retinoids or restoration of ALDH1A2 function.

## Results

### Clinical outcome of ALDH1A2^high^ tumors depends on the presence of CRABP2

**Fig. 1:**
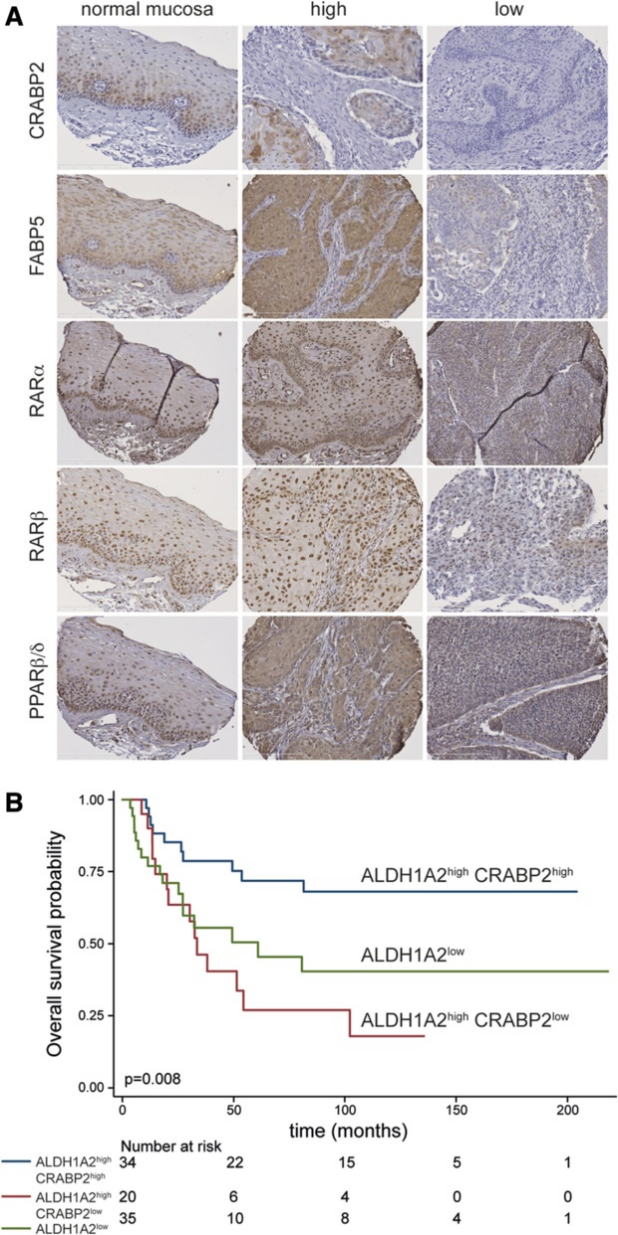
Favorable survival of ALDH1A2^high^ OPSCC depends on the presence of CRABP2. **a** Representative pictures of IHC stained tissue sections demonstrate heterogeneous expression (brown signal) of key regulators of RA signaling in keratinocytes of normal mucosa (left panel) and tumor cells of primary OPSCC (middle and right panel). Counterstaining with hematoxylin to visualize tissue architecture; white bar = 200 μm. **b** Association between subgroups with ALDH1A2^high^CRABP2^high^ (blue line), ALDH1A2^high^CRABP2^low^ (red line) and ALDH1A2^low^ (green line) staining pattern and overall survival was assessed by univariate Kaplan-Meier analysis. Number at risk indicates the total amount of patients per subgroup, which were alive and not censored at the indicated time points and were considered to calculate the overall survival probability. *P* values were calculated by log-rank test

To address the question, whether improved clinical outcome of ALDH1A2^high^ tumors is related to the presence of key components of RA signaling, immunohistochemical staining was conducted on serial sections of tissue microarrays consisting of samples from primary OPSCC for which ALDH1A2 protein levels were determined previously [8]. Prominent nuclear staining of the intracellular transporters CRABP2 and FABP5, as well as nuclear receptors RARα, RARβ and PPARβ/δ were detected in basal and supra-basal keratinocytes of normal mucosa, while a more heterogeneous expression pattern concerning staining intensity and intracellular localization of all five proteins was found in tumor cells (Fig. 1a). The expression patterns of the individual proteins were evaluated according to the relative amount of positive tumor cells and the corresponding staining intensity, and investigated with respect to their prognostic value on overall survival (OS). Univariate Kaplan-Meier analysis revealed a significant better OS for CRABP2^high^ as compared to CRABP2^low^ as well as for FABP5^high^ as compared to FABP5^low^ protein levels, respectively (Additional file 1: Figure S1A-B). In contrast, no significant correlation was found for RARα, RARβ or PPARβ/δ protein levels and OS (Additional file 1: Figure S1C-E). Whilst protein levels of CRABP2 and FABP5 correlated with OS, their expression was not associated with any clinical or pathological feature tested (Additional file 2: Table S3-4). Integrative subgroup analysis showed that ALDH1A2^high^ tumors with CRABP2^high^ expression had a significantly higher probability for improved OS, which was independent of the FABP5 staining pattern (Additional file 1: Figure S1F). In contrast, ALDH1A2^high^ tumors in the absence of CRABP2 had a poor prognosis similar to ALDH1A2^low^ tumors (Fig. 1b). Multivariate Cox regression models confirmed that the combined staining pattern of ALDH1A2^high^CRABP2^high^ served as an independent predictor for PFS and OS of OPSCC patients, when adjusted for gender, age, clinical stage, tobacco and alcohol consumption, type of first-line therapy and HPV status (Table 1). In summary these data strongly supported the assumption that improved clinical outcome of ALDH1A2^high^ OPSCCs critically depends on CRABP2-related RA metabolism and signaling.

**Table 1:** Multivariate Cox regression analysis for OS and PFS

	Overall survival			Progression-free survival		
Risk factor	HR	95 % CI	*p*-value	HR	95 % CI	*p*-value
Gender female vs male	1.016	0.47–2.19	0.967	0.960	0.48–1.94	0.911
Age	0.966	0.49–1.89	0.921	0.862	0.45–1.64	0.650
>57 vs ≤57						
Clinical stage	5.699	1.35–24.09	0.018	3.353	1.15–9.77	0.027
III/IV vs I/II						
Tobacco current vs. never	0.687	0.15–3.15	0.629	0.431	0.12–1.49	0.184
Tobacco former vs. never	1.261	0.23–6.88	0.789	0.877	0.21–3.60	0.856
Alcohol current vs never	0.786	0.28–2.18	0.644	1.261	0.50–3.16	0.621
Therapy^1^ radiochemo vs surgery	0.953	0.46–1.98	0.897	0.798	0.40–1.60	0.524
HPV status^2^ related vs non-related	0.404	0.17–0.96	0.041	0.305	0.13–0.70	0.005
Subgroup	0.303	0.14–0.63	0.002	0.395	0.20–0.77	0.007
ALDH1A2^high^CRABP2^high^ vs others						

*HR = Hazard ratio, CI = confidence interval,* ^*1*^ *first line treatment,* ^*2*^ *related = viral DNA* ^*+*^ *RNA* ^*+*^ *, non-related = viral DNA* ^*+*^ *RNA* ^*−*^ *or viral DNA* ^*−*^ *according to* [32]

### ATRA treatment impairs clonogenic growth of ALDH1A2-negative Cal27 cells

**Fig. 2:**
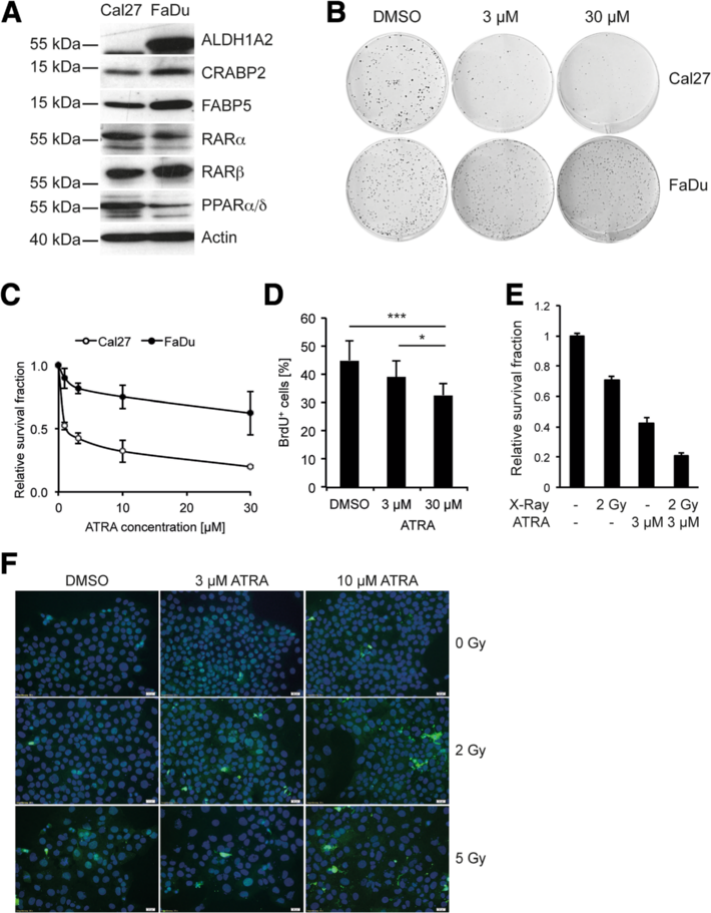
ALDH1A2 expression in HNSCC cell lines and impact of ATRA on tumor-related processes. **a** Western blot analysis with whole cell lysate demonstrates protein expression of ALDH1A2 and key regulators of RA signaling in FaDu and Cal27 cells. Detection of β-Actin served as control for quantity and quality of protein lysates. **b** Representative pictures of a colony-forming assay with Cal27 (upper panel) and FaDu cells (lower panel), which were cultured in the presence of ATRA at the indicated concentrations or DMSO as control. **c** The graph represents the relative survival fraction of Cal27 and FaDu cells following cultivation at the indicated concentration of ATRA. Data represent mean values ± SD of three independent experiments. **d** Relative number of BrdU-positive Cal27 cells following treatment with indicated concentrations of ATRA or DMSO as control. **e** Relative survival fraction of Cal27 cells after single irradiation with a dose of 2 Gy with or without ATRA treatment (3 μM). Bars represent mean values + SD of three independent replicates. * *p*-value ≤ 0.05, *** *p*-value ≤ 0.0005. **f** Representative pictures of an immunofluorescence staining for cleaved caspase 3 (green signal) with Cal27 cells, which were treated with indicated concentrations of ATRA or DMSO as control with or without irradiation (2 Gy or 5 Gy). Nuclear staining was done with H33324 (blue signal)

The data presented so far proposed a model in which patients with ALDH1A2^low^ tumors, who are at high risk for treatment failure might benefit from restoration of RA availability as long as their tumor cells express CRABP2. To further support this model, several human HNSCC cell lines were screened by Western blot analysis and prominent ALDH1A2 expression was found in FaDu, UMSCC, Detroit 562, SCC25, Lau2068, Lau2104 and Lau2105, but not Cal27, Lau2073 and Lau2081 cell lines (Fig. 2a and Additional file 3: Figure S2A-B). FaDu and Cal27 cells were selected for further analysis as both cell lines expressed similar amounts of CRABP2 and other components of RA signaling, but differed in ALDH1A2 expression. HPLC analysis revealed a two-fold higher concentration of intracellular RA levels in FaDu as compared to Cal27 cells and treatment with the ALDH1A2 inhibitor WIN18.446 reduced relative RA levels almost two-fold in FaDu but not Cal27 cells (Additional file 3: Figure S2C-D), supporting a positive association between the presence of ALDH1A2 and RA metabolism.

Next, the sensitivity of FaDu and Cal27 cells to activation of RA signaling was determined by a colony-forming assay (CFA). Both cell lines were treated every second day with all-trans retinoic acid (ATRA) and their clonogenic growth was monitored after two weeks (Fig. 2b-c). A concentration dependent decrease in the survival fraction for both cell lines was observed. However, ALDH1A2-negative Cal27 cells exhibited a more pronounced response (effective dose ED50 ≈ 1 μM) as compared to FaDu cells (ED ≈ 44 μM). A similar trend was also observed after treatment with Adapalene or Fenretinide, respectively, which belong to the new generation of synthetic retinoids (Additional file 3: Figure S2E-F). Similar to FaDu cells, ALDH1A2-positive Detroit562 and UMSCC-17B cells exhibited a higher resistance to ATRA administration in a colony-forming assay (Additional file 4: Figure S3B). Impaired clonogenic growth of Cal27 cells after ATRA treatment was consistent with reduced proliferation as determined by BrdU incorporation (Fig. 2d), which was not found for ALDH1A2-positive cell lines tested (Additional file 4: Figure S3A). Furthermore, treatment with 3 μM ATRA sensitized Cal27 cells to irradiation at a dose of 2 Gy, but had no or only a minor effect on ALDH1A2-postitive cells (Fig. 2e and Additional file 4: Figure S3B). The radiosensitization of Cal27 cells by ATRA was associated by a concentration dependent increase in cleaved caspase 3 levels, indicating accelerated apoptosis by the combined treatment (Fig. 2f). These data provided further experimental evidence that loss ALDH1A2 expression could serve as surrogate marker for HNSCC patients, who might benefit from a treatment with retinoids.

### Inhibition of ALDH1A2-RAR signaling induces loss of cell adhesion and gain of a mesenchymal-like phenotype

**Fig. 3:**
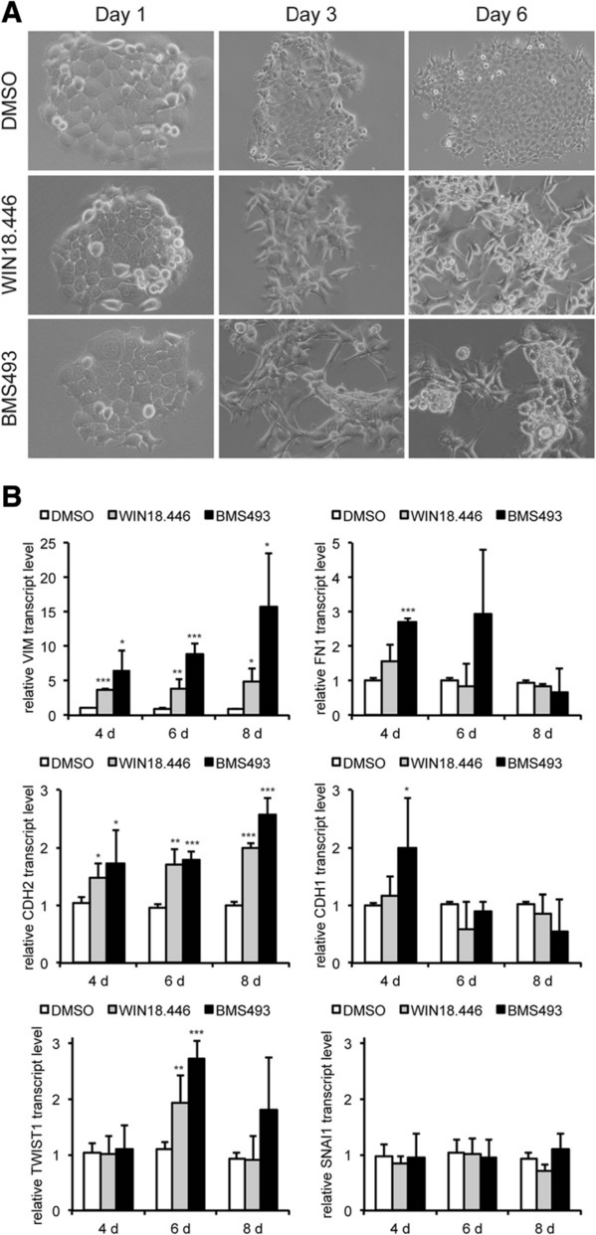
Pharmacological inhibition of ALDH1A2-RAR signaling induces loss of cell-cell junctions and a mesenchymal-like phenotype. **a** Representative brigth field pictures of FaDu cells, which were treated with DMSO, 3 μM WIN18.446 or 3 μM BMS493 for the indicated time points. **b** FaDu cells were treated as described in (**a**) for the indicated time points and relative transcript levels of vimentin (*VIM*), fibronectin (*FN1*), N-cadherin (*CDH2*), E-cadherin (*CDH1*), Twist (*TWIST1*) and Snail (*SNAI1*) were assessed by quantitative RT-PCR using the ∆∆CT method. Transcript levels of *ACTB* were determined as a reference and bars represent mean value + SD of two independent experiments with three replicates. *p-value < 0.05, **p-value < 0.005, ***p-value < 0.0005

To address the mode of action, of how reduced ALDH1A2-RAR signaling impacts tumor-related processes, FaDu cells were cultured in the presence of the ALDH1A2 inhibitor WIN18.446 or BMS493, an inverse agonist of pan-RAR, which induces nuclear co-repressor interaction with RARs. Control treated cells exhibited an epithelial morphology and formed well-defined clusters with tight cell-cell junctions (Fig. 3a, upper row). In contrast, inhibitor-treated cells showed loss of cell-cell junctions and a scattered phenotype within 3 days, and after 6 days in culture with the inhibitors, FaDu cells detached and developed spheroid-like structures (Fig. 3a, middle and lower row). It is worth noting that the severity of the phenotype was positively correlated with the concentration of both inhibitors (data not shown) and was more prominent for BMS493. A comparable phenotype upon WIN18.446 or BMS493 administration was also observed with Detroit562 cells (Additional file 5: Figure S4A-B), demonstrating that morphological alterations after inhibition of ALDH1A2-RAR signaling are not restricted to FaDu cells. However, UMSCC-17B cells remained epithelial-like and did not show any sign of cell scattering, despite prominent ALDH1A2 and RAR protein expression (Additional file 5: Figure S4A), suggesting a context-dependent response.

In line with the mesenchymal-like morphology both inhibitors revealed in FaDu cells a significant up-regulation of vimentin (*VIM*) and N-cadherin (CDH2) transcript levels as compared to DMSO-treated controls (Fig. 3b). Additionally, BMS493-treated cells showed a transient increase in fibronectin (*FN1*). However, no significant down-regulation in E-cadherin (*CDH1*) transcript levels was detected indicating that inhibition of ALDH1A2-RAR signaling resulted in only a partial epithelial-to-mesenchymal transition. We also determined transcript levels of Snail (*SNAI1*) and Twist (*TWIST1*), two key transcription factors in the induction and maintenance of EMT, in control and inhibitor treated FaDu cells. While no major difference was found for Snail, a significant but transient increase of Twist was detected with both inhibitors at day 6 (Fig. 3b).

A mesenchymal-like phenotype in epithelial tumor cells is often associated with higher cell motility. Accordingly, we conducted a scratch wounding assay with control, WIN18.446 and BMS493 treated Detroit562 and FaDu cells. As expected, a clear trend towards accelerated migration was found for both inhibitors as compared to control treated cells, which reached statistical significance for WIN18.446 at 36 h (Additional file 5: Figure S4C). Similar data were obtained for FaDu cells (Additional file 5: Figure S4D).

### Silencing of ALDH1A2 expression accelerates tumor growth in vivo

**Fig. 4:**
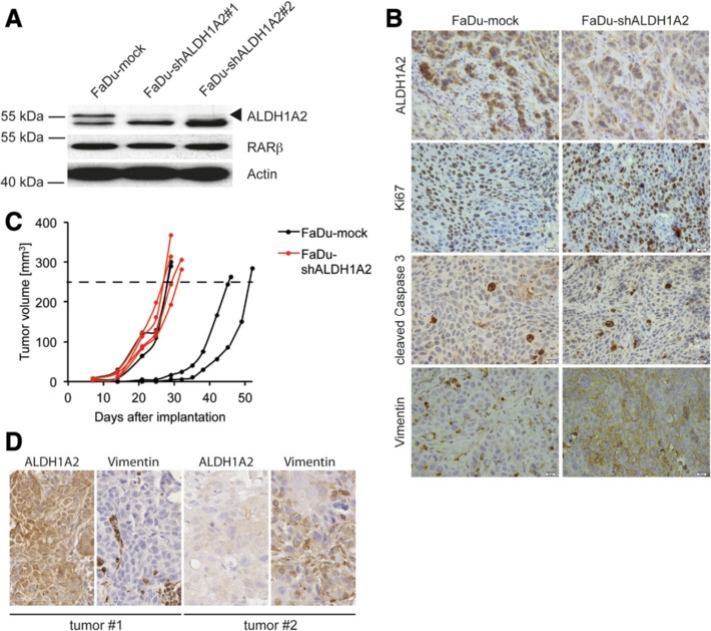
Impact of stable ALDH1A2 silencing in an orthotopic mouse xenograft model. **a** Western blot analysis with whole cell lysate of FaDu-mock and FaDu-shALDH1A2 clones confirms stable silencing of ALDH1A2, while no alteration in RARβ protein level was detected. Detection of β-Actin served as control for quantity and quality of protein lysates. **b** Representative pictures of IHC staining with tumor sections derived from FaDu-mock or FaDu-shALDH1A2-injected xenografts to analyze ALDH1A2 expression, tumor cell proliferation (Ki67), apoptosis (cleaved caspase 3) and the mesenchymal-like phenotype (Vimentin). Counterstaining with hematoxylin to visualize tissue architecture; scale bar = 20 μm. **c** The graph represents quantification of the tumor volume (in mm^3^) in mice (*n* = 4 per group) at the indicated time points after implantation with either FaDu-mock or FaDu-shALDH1A2 clones. Dashed line indicates surgical threshold. Mean values ± SD and *p* values are given in Additional file 2: Table S5. **d** Representative pictures of an IHC staining (brown signal) with serial tumor sections demonstrate inverse expression of ALDH1A2 and vimentin in OPSCC. Counterstaining with hematoxylin to visualize tissue architecture; white bar = 80 μm

Finally, the impact of ALDH1A2 loss-of-function on tumor growth was investigated in vivo by orthotopic injection of stable FaDu-shALDH1A2 or FaDu-mock control cells in the floor of the mouth of nude mice. shRNA-mediated silencing of ALDH1A2 protein expression was confirmed in vitro by Western immunoblot analysis with whole-cell lysate and in vivo by immunohistochemical staining on tumor sections (Fig. 4a-b). Tumor incidence was comparable for both cell lines (4 out of 5 animals), but FaDu-shALDH1A2 exhibited an accelerated tumor growth, which was statistical significant 25 days upon injection (Fig. 4c and Additional file 2: Table S5). While tumor growth was rather homogeneous in the subgroup of FaDu-shALDH1A2 implanted mice and all tumors reached the surgical threshold within 29 days, 50 % of FaDu-mock tumors reached a tumor volume of 250 mm^3^ between 46–52 days. Accelerated tumor growth was not related to an obvious increase of Ki67-positive or a decrease in cleaved caspase 3-positive tumor cells indicating no major difference in tumor cell proliferation or apoptosis in vivo (Fig. 4b). However, tumors derived from FaDu-shALDH1A2 cells were characterized by prominent vimentin expression, which was not detected for tumors from FaDu-mock controls, and was in line with the mesenchymal-like phenotype in vitro (Fig. 4b). Consistent with this result an inverse expression of ALDH1A2 and vimentin was also evident in primary OPSCC by immunhistochemical staining of serial tumor section (Fig. 4d).

## Discussion

In the past, gene promoter hyper-methylation and subsequent low ALDH1A2 expression was identified as a common feature for OPSCC and served as a risk factor for an unfavorable prognosis [8]. Reduced ALDH1A2 levels were reported previously in tumor cell lines, a mouse tumor model and tumor specimens of prostate cancer patients [17, 18]. In these studies low ALDH1A2 expression was explained, at least in part, by aberrant DNA methylation. Furthermore, high ALDH1A2 transcript levels correlated with improved overall survival of breast cancer patients [19]. Although such data point to a putative tumor suppressor function of ALDH1A2 in the pathogenesis of several epithelial cancers, the causal link between loss of ALDH1A2 function and molecular mechanisms of treatment failure have not been addressed so far.

ALDH1A2 catalysis an irreversible step in the synthesis of RA and thereby regulates distinct aspects of cell proliferation, differentiation and apoptosis under physiological and pathological conditions [11, 12]. The anti-tumorigenic activity of natural and synthetic retinoids on cancer cells has been established in numerous in vitro and in vivo models for distinct tumor entities [13, 15]. Accordingly, it is worth speculating that the detrimental effect of reduced ALDH1A2 expression on malignant progression and prognosis is due to impaired RA-dependent signaling. This assumption is strongly supported by our data demonstrating improved clinical outcome of ALDH1A2^high^ OPSCC only in the presence of CRABP2, and an overlapping phenotype of ALDH1A2-positive FaDu and Detroit562 cells after pharmaceutical inhibition of either ALDH1A2 activity or RAR-dependent transcription.

Inhibition of ALDH1A2-RAR signaling in vitro revealed a mesenchymal-like phenotype, which was characterized by loss of cell-to-cell adhesion, accelerated migration and induced expression of markers indicating induction and maintenance of epithelial-to-mesenchymal transition, such as vimentin, N-cadherin, fibronec tin and Twist [20, 21]. The inverse correlation between ALDH1A2 and vimentin expression in tumor cells was further supported in a mouse xenograft model in vivo and tumor sections from OPSCC patients. Although, the underlying molecular mechanism remains to be elucidated, several studies reported that ATRA inhibits tumor cell invasion and metastatic potential in diverse model systems by modulating cell-to-cell adhesion and RAR-dependent regulation of proteins implicated in epithelial-to-mesenchymal transition [22–25]. It is also worth noting that a continuous cultivation of FaDu cells in the presence of WIN18.446 or BMS493, respectively, results in formation of stable and expandable tumorspheres, suggesting that tumor cells lacking ALDH1A2-RAR signaling also gain the capacity to avoid anoikis and acquire stem cell traits (unpublished data). However, the reduced cell-matrix adhesion upon inhibition of ALDH1A2-RAR signaling could cause a certain bias concerning the interpretation of accelerated migration in 2D as it is quite likely that a substantial amount of inhibitor-treated cells were lost over time by cell detachment. In line with this assumption, administration of higher amounts of WIN18.446 (data not shown) or the more potent inhibitor BMS493 did not improve cell motility, despite increased cell scattering and a more prominent mesenchymal-like phenotype. This raises the attractive question, whether HNSCC cells in the absence of ALDH1A2-RAR signaling gain the capacity of single cell and amoeboid-like migration, which should be addressed in more sophisticated models of 3D migration in future studies. Furthermore, it will be a major challenge to unravel critical downstream targets as well as relevant interactions between ALDH1A2-RAR signaling and other key regulators of mesenchymal transition as well as stem cell traits, especially those that have been shown to correlate with tumor cell dissemination and treatment failure of HNSCC patients [20, 21].

## Conclusions

Based on these findings, we speculate that a subgroup of HNSCC patients at high risk for treatment failure under currently established therapeutic regimens might benefit from an adjuvant treatment with retinoids to restore ALDH1A2-RAR signaling in tumor cells. Administration of retinoids as chemopreventive strategy for premalignant lesions or as a therapeutic option for HNSCC was tested in several clinical trials [26–30]. Despite initial encouraging results this class of compounds was not translated into clinical practice due to intrinsic resistance to retinoids, the toxic profile and lack of reliable biomarkers to predict treatment responders [31]. Meanwhile, new compounds with improved pharmacology and reduced toxicity are available and the lack of ALDH1A2 expression could serve as an attractive biomarker to stratify HNSCC patients, who might benefit from an adjuvant treatment with these new synthetic retinoids.

## Methods

### Patient samples and tissue microarray

Tumor specimens for this retrospective study were obtained from oropharyngeal squamous cell carcinoma (OPSCC) patients, who were treated at the University Hospital Heidelberg between 1990 and 2008. Paraffin-embedded tissue specimens were provided by the tissue bank of the National Center for Tumor Disease (Institute of Pathology, University Hospital Heidelberg) after approval by the local institutional review board (ethic vote: 206/2005). The study was performed according to the ethical standards of the Declaration of Helsinki. For all tumor samples, clinical and follow-up data were available from the Department of Otolaryngology, Head and Neck Surgery at the University Hospital Heidelberg and are listed in Additional file 2: Table S3-4. HPV16 DNA and viral transcript status for patients of the cohort was determined previously [32], and generation of tissue microarrays has been described elsewhere [33].

### Immunohistochemical staining and scoring system

Tissue microarrays and tumor sections were incubated with antibodies that are listed in Additional file 2: Table S1. Immunostaining was visualized with the TSA Amplification Kit (Perkin Elmer, Rodgau, Germany) and DAB peroxidase substrate (Vector Laboratories, Burlingame, USA) according to the manufacturers instructions. Counterstaining was done by hematoxylin to visualize tissue integrity. Specificity of antibodies used in this study to detect expression of ADL1A2 (HPA010022), CRABP2 (HPA004135) and RARβ (HPA004174) was confirmed by similar staining patterns comparing experimental data on our TMAs and those provided by www.proteinatlas.org. Staining specificity of antibodies used to detect expression of FABP5 (ab84028), RARα (WH0005914M1) and PPARβ/δ (ab137724) was confirmed by IHC staining on serial TMA sections using independent antibodies, respectively (data not shown).

Bright field pictures were taken with the Nikon Eclipse Ti microscope using the Nikon Imaging Software NIS-Elements 3.20.02. Stained tissue microarrays were scanned using the Nanozoomer HT Scan System (Hamamatsu Photonics, Japan). Protein expression was evaluation by three independent observers using the NDP Viewer software (version 1.1.27) and considering the relative amount of positive tumor cells (score 1 = no positive cell, score 2 ≤ 33 %, 33 % > score 3 ≤ 66 %, score 4 > 66 %) and the staining intensity (score 1 = no staining, score 2 = weak staining, score 3 = moderate staining, score 4 = high staining) as described previously [8]. Both scores were multiplied to calculate the final immunoreactivity score (IRS, range 1–16) and the cut-off values for subgroups with high versus low protein levels was IRS = 4 for CRABP2, IRS = 6 for FABP5, IRS = 9 for RARα, RARβ, and PPARβ/δ, respectively.

### Statistical analysis

Statistical analysis was done using SPSS (version 19) and SAS (version 9.2) statistics software. Differences between the groups were assessed using Chi square test or Fisher’s exact test. Overall survival was calculated as the time from the date of first-line therapy to the date of tumor-related death within the follow-up interval (events). Survival time of patients who were alive or were dead due to causes other than OPSCC were censored. Progression-free survival was calculated from the date of primary therapy to the date of the first local recurrence, lymph node or distant metastasis, second primary carcinoma or date of tumor-related death within the follow-up period (events), or to the date of OPSCC-unrelated death or without progression (censored). The method of Kaplan–Meier was used to estimate survival distributions and differences between groups were determined by log-rank tests. A multivariate Cox proportional hazard model was used to assess the association between protein expression scores and overall or progression-free survival of cancer patients, together with the covariates age, gender, clinical stage, alcohol or tobacco consumption, first-line therapy and HPV status. The validity of the proportional hazards assumption was tested with the Supreme Test for proportional hazards assumption and was met for all covariates. In all statistical tests, a *p*-value of 0.05 or below was considered as statistically significant.

### Cell culture experiments

Human cell lines SCC25, FaDu and Cal27 were purchased from ATCC (http://www.lgcstandards-atcc.org/), Detroit 562 from CLS (Cell Lines Service GmbH, Eppelheim, Germany) and UMSCC-17B were kindly provided by Dr. I. Tinhofer (Charité Universitätsmedizin Berlin, Germany). Lau2068, Lau2073, Lau2081, Lau2104 and Lau2105 were established from tumors of HNSCC patients, who were treated at the University Hospital Lausanne, Switzerland. All tumor cell lines were maintained in Dulbecco’s Modified Eagle’s Medium (Sigma, Germany) supplemented with 10 % fetal bovine serum (Invitrogen, Germany), 2 mM L-Glutamine (Invitrogen, Germany) and 50 μg/ml Penicillin-Streptomycin (Invitrogen, Germany) in a humidified atmosphere of 6 % CO2 at 37 °C. Authentication of commercial available cell lines was confirmed by the Multiplex Human Cell Line Authentication Test (Multiplexion, Germany).

For colony formation assays 300–1,000 FaDu, Cal27, Detroit562 or UMSCC-17B cells were seeded per 6-well plate. Cells were treated with the indicated concentration of ATRA (Sigma Aldrich, Germany), Adapalene (Santa Cruz Biotechnology, US) or Fenretinide (Tocris bioscience, UK) every second day for two weeks. To determine the impact of ATRA on radiosensitivity, FaDu, Cal27, Detroit562 or UMSCC-17B cells were treated with the indicated concentration of ATRA or DMSO as control and irradiation was done once with a dose of 2 Gy (X-RAD 320, Precision X-Ray, North Branford, CT USA). After two weeks colonies were PFA-fixed and the number of colonies was determined after crystal violet staining as described in [34]. The survival fraction was computed according to [35].

Cell proliferation of control (DMSO) and ATRA treated (3 and 30 μM) tumor cells was determined by a BrdU incorporation assay as described previously [20].

FaDu, Detroit 562 and UMSCC-17B cells were cultured in the presence of either WIN18.446 (Santa Cruz Biotechnology, US) or BMS493 (Sigma Aldrich, Germany) at the indicated concentrations with medium exchange and inhibitor administration every two to three days. DMSO-treated cells served as control. Bright field and phase contrast pictures were taken with the Nikon Eclipse Ti microscope using the Nikon Imaging Software NIS-Elements 3.20.02. Fluorescence staining of the actin cytoskeleton with Phalloidin-Alexa Fluor 488 (Invitrogen, Germany) and nuclear staining with Hoechst 33342 (Calbiochem Merck, Germany) was done as described elsewhere [36].

The scratch wounding assay with Detroit562 and FaDu cells was done as described elsewhere [37].

### Protein isolation and Western blot analysis

Whole cell protein lysate was extracted using RIPA (Radioimmunoprecipitation assay) buffer [38] and protease and phosphatase inhibitor cocktails (Sigma-Aldrich). 20 μg of denatured protein were separated by Sodiumdodecylsulfate-polyacrylamide gel electrophoresis (SDS-PAGE) and transferred to polyvynil difluoride (PVDF) membranes (Millipore, Germany). After blocking with 5 % milk (Roth, Germany), membranes were incubated with primary and horseradish peroxidase coupled-secondary antibodies, which are listed in Additional file 2: Table S2. Membranes were incubated in enhanced chemiluminiscence solution (Thermo Scientific, Germany) and developed with a classic E.O.S. developer (Agfa, Germany).

### Quantification of intracellular RA levels by HPLC

Intracellular RA levels were measured as previously described [39, 40]. Briefly, 5×10^6^ cells were re-suspended in 500 μl of 200 mM acetic acid and distributed by passing 20 times through a needle of a syringe. To an aliquot (150 μl) of this cell suspension, 200 pmol of the synthetic retinoid Acitretin (25 μl; 20 μM in 100 % ethanol, Sigma Aldrich) was added as an internal standard. Two volumes (320 μl) ice-cold 100 % ethanol was added and samples were vortexed briefly. Hexane (2.5 volume; 1200 μl) was added to each sample and carefully vortexed for 1 min followed by centrifugation (5000 rpm, 20 min; 4 °C). The upper, organic, phase was then transferred to a clean glass tube and dried by centrifugal evaporation (30 °C; 40 min). The resulting residue was dissolved in methanol/acetonitrile (50:50) + 0.1 % acetic acid (30 μl), then diluted to a final volume of 100 μl with 10 mM ammonium acetate + 0.1 % acetic acid, and transferred to an amber HPLC vial and analysed. The RA analytes were separated by HPLC (Hitachi Chromaster HPLC, VWR International GmbH, Germany) using Zorbax Eclipse XDB C18 columns (Agilent Technologies, US). The column was eluted with 10 mM ammonium acetate + 0.1 % acetic acid and acetonitrile + 0.1 % acetic acid (20:80 %) with a linear gradient over 15 min to 100 % acetonitrile + 0.1 % acetic acid. The flow was 0.25 ml/min and column temperature at 30 °C. The RA analytes were detected by absorbance monitoring at 385 nm. The concentration on RA analytes was determined from serial dilutions (10–1000 pmol) of ATRA spiked into artificial plasma (150 μl), which were then processed as described. Calibration curves were constructed by plotting the peak area ratio of the RA analytes and internal standard (Acitretin) against the respective concentration. The results were normalized by total protein concentration of cell lysates. Protein concentrations were measured with the BCA Protein Assay Kit (Pierce Biotechnology, US).

### RNA extraction, cDNA synthesis and RT-PCR analysis

Total RNA from tumor cell lines was isolated with the RNeasy Mini Kit (Qiagen, Germany) following the manufacturer’s instruction. Genomic DNA digestion was performed with RNase-free DNAse Set (Qiagen, Germany). Quantity and quality of isolated RNA was determined with the help of the Nanodrop Spectrophotometer ND-1000 (peqlab, Germany). cDNA synthesis and quantitative RT-PCR was performed as described previously [41]. Annealing temperatures and sequences of all primers are listed in Additional file 2: Table S2. The cycle of threshold (CT) of the gene of interest was standardized to the CT value of the reference gene using the ∆∆CT method. For each primer pair, primer efficiency was determined by a dilution series from 0.01 to 100 ng of a cDNA mix of reference samples. A primer efficiency of 1.8 to 2.0 was accepted for further analysis.

### Generation of stable FaDu clones

FaDu cells were transfected with either a control pRS vector encoding a non-effective Hush 29-mer scrambled shRNA cassette (TR30012, OriGene Technologies, USA) or a pRS-shALDH1A2 plasmid (TR306766C, OriGene Technologies, USA) using FuGene HD Transfection Reagent (Promega, Germany) according to the manufacturer’s instruction. Following selection with 1 μg/ml Puromycin (Gibco life technologies, Germany) for one week, stable clones were established and efficient silencing of ALDH1A2 expression was confirmed on protein level.

### Mouse xenograft model

Animal experiments were approved by the Vaud Cantonal Veterinary Office and conducted in accordance with guidelines of the Ethics Committee for Animal Experimentation of the Swiss Academy of Medical Sciences. Athymic female 8-week-old NMRI-nu mice were purchased from JANVIER LABS (Le Genest-Saint-Isle, France). Mice were housed in groups of 5 and had access to water and food *ad libitum* at the University of Lausanne (UNIL) animal facility. 8×10^5^ cells in a 30 μl HBSS cell suspension were injected with 20 μl Matrigel (BD Biosciences) into the subcutaneous tissue of the anterior floor of mouth approximately 3 mm caudal to the mandible under isoflurane anesthesia. Tumor size was measured by a caliper and calculated as V = (L×W^2^)/2 twice a week. During the time period of tumor growth no animal showed cachexia (weight loss > 15 %) or any signs of behavioral disturbance. When the tumor volume reached a maximum size of 250 mm^3^, mice were euthanized by CO_2_ inhalation, and tumor samples were divided into fragments which were snap-frozen in liquid nitrogen for molecular analyses or fixed with 4 % paraformaldehyde and embedded with paraffin for histological analyses.

## Additional files

Additional file 1: Figure S1. Association between subgroups with high or low protein expression of CRABP2 (**A**), FABP5 (**B**), RARα (**C**), RARβ (**D**) or PPARα/δ (**E**) and overall survival of OPSCC patients was assessed by univariate Kaplan-Meier analysis. (**F**) Kaplan-Meier analysis demonstrates overall survival probability for subgroups with indicated staining patterns for ALDH1A2, CRABP2 and FABP5. Number at risk indicates the total amount of patients per subgroup, which were alive and not censored at the indicated time points and were considered to calculate the overall survival probability. *P* values were calculated by log-rank tests. (TIF 1257 kb) Additional file 2:
**Table S1.** List of primary and secondary antibodies. **Table S2.** List of primer sequences for RT-PCR analysis. **Table S3.** Correlation analysis for CRABP2 protein levels and clinical or pathological features of the OPSCC cohort. **Table S4.** Correlation analysis for FABP5 protein levels and clinical or pathological features of the OPSCC cohort. **Table S5.** Tumor volume (in mm^3^) ± SD in mice (*n* = 4 per group) after injection with either FaDu-mock or FaDu-shALDH1A2 clones. (DOCX 59 kb) Additional file 3: Figure S2. ALDH1A2 expression in HNSCC cell lines and morphological phenotype upon inhibition of ALDH1A2-RAR signaling. Western blot analysis with whole cell lysate demonstrates protein expression of ALDH1A2 and key regulators of RA signaling in UMSCC-17B, Detroit562, FaDu, Cal27 and SCC25 cells (**A**), and ALDH1A2 protein levels in newly established HNSCC cell lines from Lausanne (**B**). Detection of β-Actin served as control for quantity and quality of protein lysates. Relative RA levels were determined by HPLC analysis with whole cell lysate of untreated (**C**), and DMSO-treated control or WIN18.446-treated Cal27 and FaDu cells (**D**). Bars represent mean values ± SD of three independent replicates. Graphs indicate relative survival fraction of Cal27 and FaDu cells, which were treated with the indicated concentration of Adapalene (**E**) or Fenretinide (**F**). Data represent mean values ± SD of three independent replicates. (TIF 301 kb) Additional file 4: Figure S3. (**A**) Relative number of BrdU-positive FaDu, Detroit562 and UMSCC-17B cells following treatment with indicated concentrations of ATRA or DMSO as control. Bars represent mean values + SD of three independent replicates. (**B**) Relative survival fraction of Cal27, FaDu, Detroit562 and UMSCC-17B cells after single irradiation with (red line) or without (blue line) a dose of 2 Gy in combination with ATRA treatment (1–30 μM). (TIF 899 kb) Additional file 5: Figure S4. (**A**) Representative phase contrast pictures of FaDu, Detroit562 and UMSCC-17B cells, which were treated with DMSO, 3 μM WIN18.446 or 3 μM BMS493 for four days. (**B**) Representative fluorescent pictures of FaDu and Detroit562 cells, which were treated as described in (**A**), and were stained with Phalloidin-Alexa488 (green signal). Nuclear staining was done with H33342 (blue signal). Migration of Detroit562 (**C**) and FaDu cells (**D**), which were treated with DMSO (white bars), 3 μM WIN18.446 (grey bars) or 3 μM BMS493 (black bars), in a scratch wounding assay was determined by the relative gap closure at the indicated time points. * *p* value ≤ 0.05. (TIF 4129 kb)
