# Supplementary figures and images for: Impaired aldehyde dehydrogenase 1 subfamily member 2A-dependent retinoic acid signaling is related with a mesenchymal-like phenotype and an unfavorable prognosis of head and neck squamous cell carcinoma
Source: Mol Cancer. 2015 Dec 3;14:204. doi: 10.1186/s12943-015-0476-0 (PMC4669670; doi:10.1186/s12943-015-0476-0)

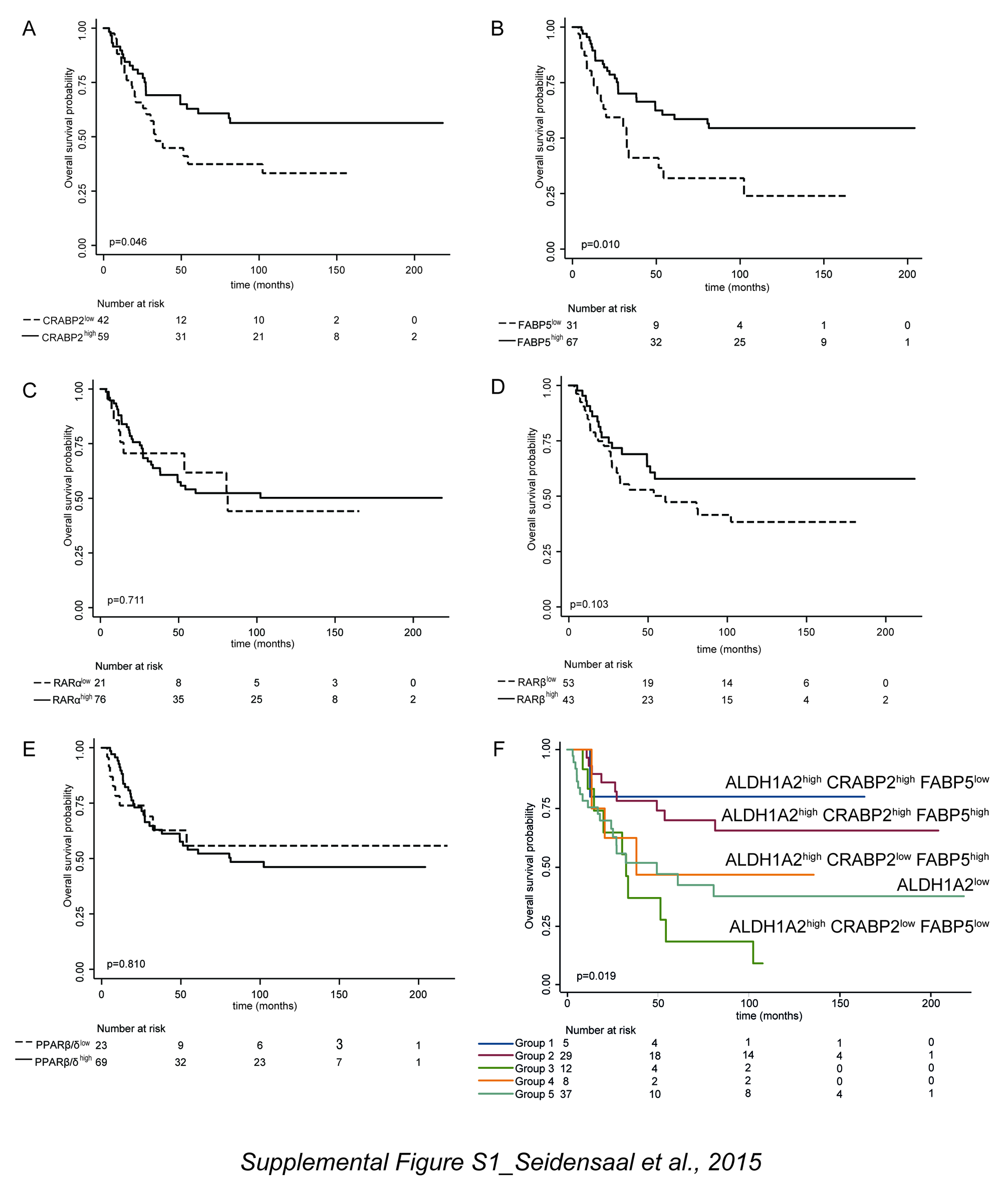

Supplement: Additional file 1: Figure S1. — Association between subgroups with high or low protein expression of CRABP2 (A), FABP5 (B), RARα (C), RARβ (D) or PPARα/δ (E) and overall survival of OPSCC patients was assessed by univariate Kaplan-Meier analysis. (F) Kaplan-Meier analysis demonstrates overall survival probability for subgroups with indicated staining patterns for ALDH1A2, CRABP2 and FABP5. Number at risk indicates the total amount of patients per subgroup, which were alive and not censored at the indicated time points and were considered to calculate the overall survival probability. P values were calculated by log-rank tests. (TIF 1257 kb) [file 12943_2015_476_MOESM1_ESM.tif]

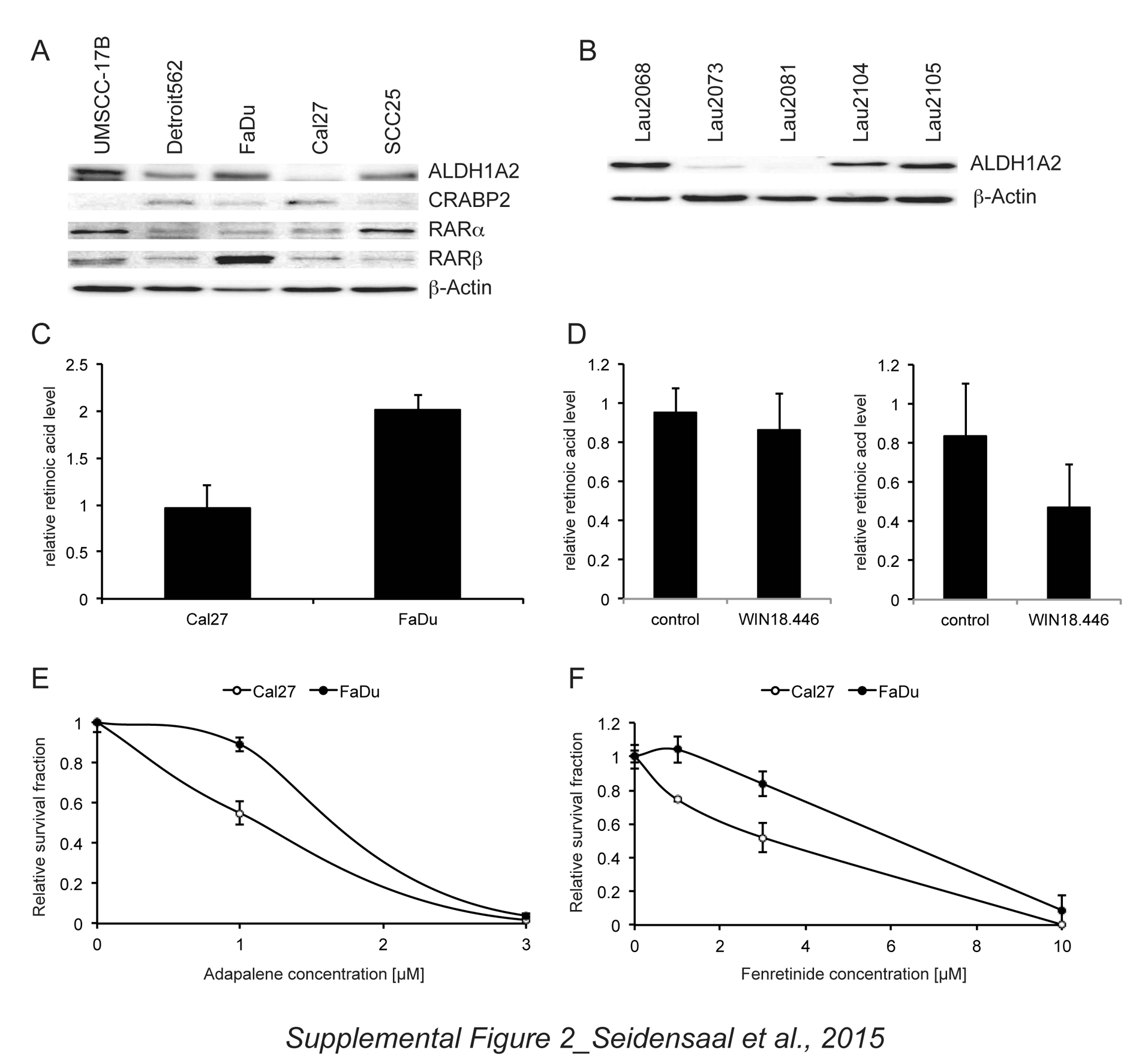

Supplement: Additional file 3: Figure S2. — ALDH1A2 expression in HNSCC cell lines and morphological phenotype upon inhibition of ALDH1A2-RAR signaling. Western blot analysis with whole cell lysate demonstrates protein expression of ALDH1A2 and key regulators of RA signaling in UMSCC-17B, Detroit562, FaDu, Cal27 and SCC25 cells (A), and ALDH1A2 protein levels in newly established HNSCC cell lines from Lausanne (B). Detection of β-Actin served as control for quantity and quality of protein lysates. Relative RA levels were determined by HPLC analysis with whole cell lysate of untreated (C), and DMSO-treated control or WIN18.446-treated Cal27 and FaDu cells (D). Bars represent mean values ± SD of three independent replicates. Graphs indicate relative survival fraction of Cal27 and FaDu cells, which were treated with the indicated concentration of Adapalene (E) or Fenretinide (F). Data represent mean values ± SD of three independent replicates. (TIF 301 kb) [file 12943_2015_476_MOESM3_ESM.tif]

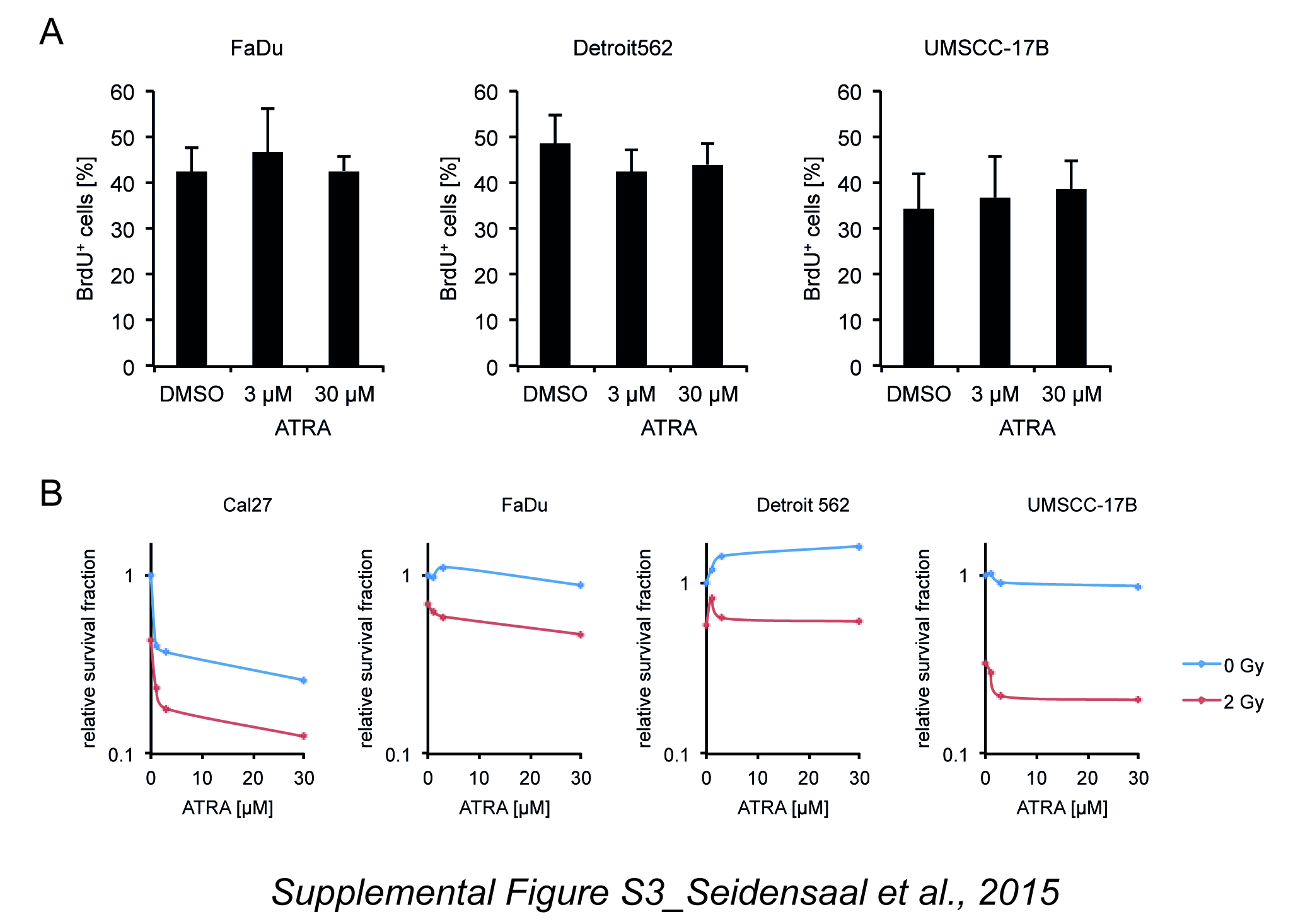

Supplement: Additional file 4: Figure S3. — (A) Relative number of BrdU-positive FaDu, Detroit562 and UMSCC-17B cells following treatment with indicated concentrations of ATRA or DMSO as control. Bars represent mean values + SD of three independent replicates. (B) Relative survival fraction of Cal27, FaDu, Detroit562 and UMSCC-17B cells after single irradiation with (red line) or without (blue line) a dose of 2 Gy in combination with ATRA treatment (1–30 μM). (TIF 899 kb) [file 12943_2015_476_MOESM4_ESM.tif]

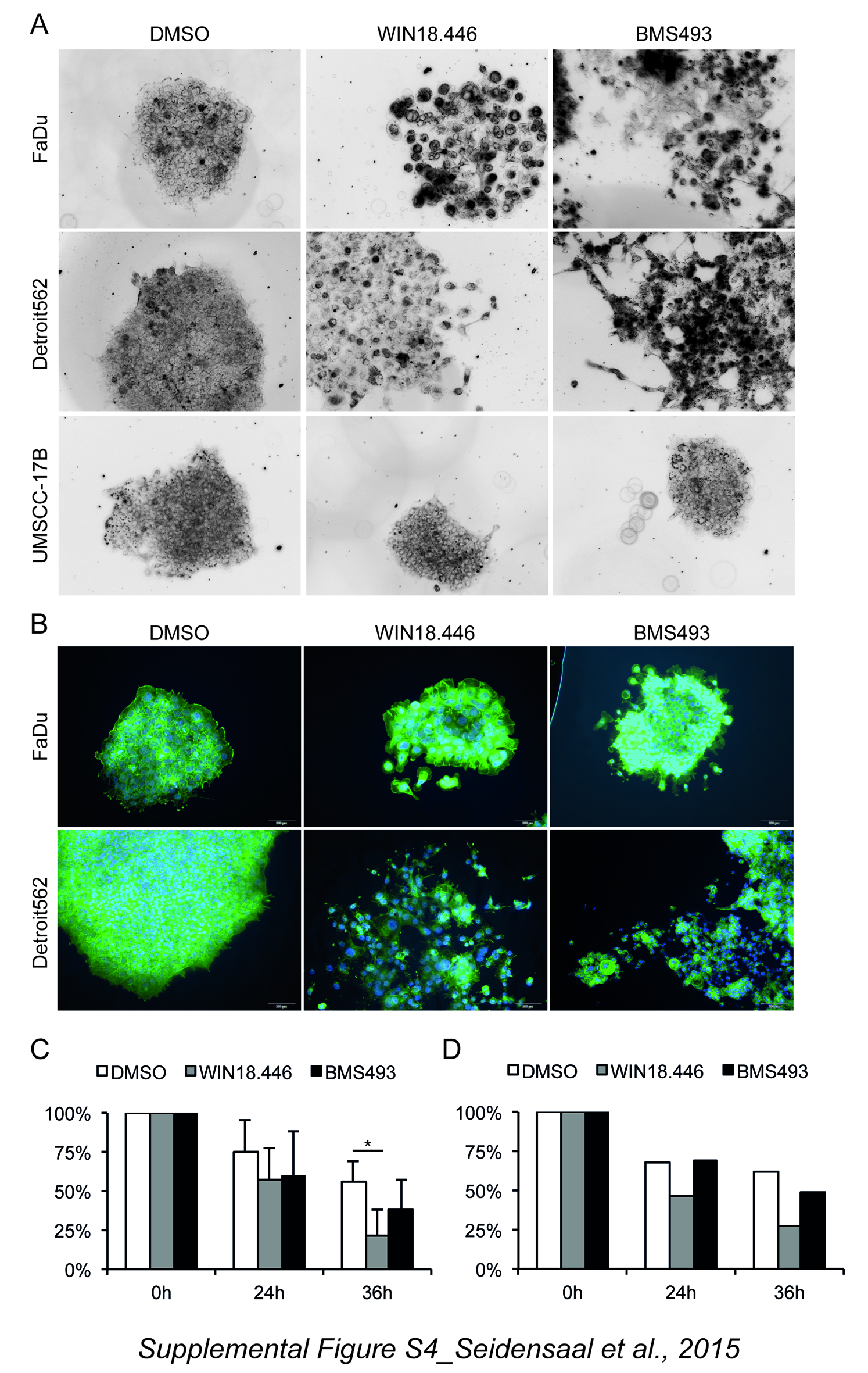

Supplement: Additional file 5: Figure S4. — (A) Representative phase contrast pictures of FaDu, Detroit562 and UMSCC-17B cells, which were treated with DMSO, 3 μM WIN18.446 or 3 μM BMS493 for four days. (B) Representative fluorescent pictures of FaDu and Detroit562 cells, which were treated as described in (A), and were stained with Phalloidin-Alexa488 (green signal). Nuclear staining was done with H33342 (blue signal). Migration of Detroit562 (C) and FaDu cells (D), which were treated with DMSO (white bars), 3 μM WIN18.446 (grey bars) or 3 μM BMS493 (black bars), in a scratch wounding assay was determined by the relative gap closure at the indicated time points. * p value ≤ 0.05. (TIF 4129 kb) [file 12943_2015_476_MOESM5_ESM.tif]
