# Supplementary material for: Impaired aldehyde dehydrogenase 1 subfamily member 2A-dependent retinoic acid signaling is related with a mesenchymal-like phenotype and an unfavorable prognosis of head and neck squamous cell carcinoma
Source: Mol Cancer. 2015 Dec 3;14:204. doi: 10.1186/s12943-015-0476-0 (PMC4669670; doi:10.1186/s12943-015-0476-0)
Supplement: Additional file 2: — Table S1. List of primary and secondary antibodies. Table S2. List of primer sequences for RT-PCR analysis. Table S3. Correlation analysis for CRABP2 protein levels and clinical or pathological features of the OPSCC cohort. Table S4. Correlation analysis for FABP5 protein levels and clinical or pathological features of the OPSCC cohort. Table S5. Tumor volume (in mm3) ± SD in mice (n = 4 per group) after injection with either FaDu-mock or FaDu-shALDH1A2 clones. (DOCX 59 kb) [file 12943_2015_476_MOESM2_ESM.docx]

**Supplemental Table S1.** List of primary and secondary antibodies

|  | Cat. No. | Clone & Species | Company | Application | Dilution |
| --- | --- | --- | --- | --- | --- |
| ALDH1A2 | HPA010022 | Polyclonal rabbit | Sigma-Aldrich | IHC  WB | 1:50-200  1:200 |
| CRABP2 | HPA004135 | Polyclonal rabbit | Sigma-Aldrich | IHC  WB | 1:250  1:250 |
| FABP5 | ab84028 | Polyclonal rabbit | Abcam | IHC  WB | 1:500  1:500 |
| RARα | WH0005914M1 | Monoclonal mouse | Sigma-Aldrich | IHC  WB | 1:150  1:1000 |
| RARβ | HPA004174 | Polyclonal rabbit | Sigma-Aldrich | IHC  WB | 1:100  1:250 |
| PPARβ/δ | ab137724 | Polyclonal rabbit | Abcam | IHC  WB | 1:150  1:500 |
| Ki67 | Ab15580 | Polyclonal rabbit | Abcam | IHC | 1:500 |
| cleaved caspase 3 | #9661 | Polyclonal rabbit | Cell Signaling | IHC | 1:100 |
| Vimentin | 61013 | monoclonal mouse | Progen | IHC | 1:100 |
| β-Actin | A5441 | AC-15; monoclonal mouse | Sigma-Aldrich | WB | 1:10000 |
| BrdU  Alexa555 | B35131 | MoBU-1; monoclonal mouse | Invitrogen | IF | 1:100 |
| anti-rabbit-HRP | #7074 | Goat | Cell Signaling | WB | 1:10000 |
| anti-mouse-HRP | #7076 | Horse | Cell Signaling | WB | 1:10000 |

*WB = Western blot, IHC = immunohistochemistry*

**Supplemental Table S2.** List of primer sequences for RT-PCR analysis

| **Gene Symbol** | | **Sequence** | **T_A_ (°C)** |
| --- | --- | --- | --- |
| *ACTB* | for | CCAACCGCGAGAAGATGA | 60 |
|  | rev | CCAGAGGCGTAGAGGGATAG |  |
| *CDH1* | for | AATCCCACCACGTACAAGGG | 60 |
|  | rev | GGTATTGGGGGCATCAGCAT |  |
| *CDH2* | for | CACCGTGGTCAAACCAATCG | 60 |
|  | rev | GGTGCTGAATTCCCTTGGCT |  |
| *FN1* | for | CTTTGGTGCAGCACAACTTC | 60 |
|  | rev | TCCTCCTCGAGTCTGAACCA |  |
| *TWIST1* | for | GCCGGAGACCTAGATGTCATT | 60 |
|  | rev | TTTTAAAAGTGCGCCCCACG |  |
| *SNAI1* | for | GAGGCGGTGGCAGACTAG | 60 |
|  | rev | GACACATCGGTCAGACCAG |  |
| *VIM* | for | CTCTGGCACGTCTTGACCTT | 60 |
|  | rev | TCCTGGATTTCCTCTTCGTG |  |

**Supplemental Table S3.** Correlation analysis for CRABP2 protein levels and clinical or pathological features of the OPSCC cohort

|  |  | **CRABP2^low^** | **CRABP2^high^** | **p-value** |
| --- | --- | --- | --- | --- |
| Age [years] | ≤58 | 24 | 27 | 0.314 |
|  | >58 | 18 | 32 |  |
| Gender | male | 28 | 45 | 0.368 |
|  | female | 14 | 14 |  |
| Tumor size | T1/2 | 16 | 22 | 1.000 |
|  | T3/4 | 26 | 36 |  |
|  | missing^1^ |  | 1 |  |
| Lymph node status | N0 | 9 | 13 | 1.000 |
|  | N+ | 33 | 45 |  |
|  | missing^1^ |  | 1 |  |
| Distant metastasis | M0 | 40 | 57 | 0.177 |
|  | M+ | 2 | 0 |  |
|  | missing^1^ |  | 2 |  |
| Clinical stage | I/II | 4 | 7 | 0.757 |
|  | III/IV | 38 | 51 |  |
|  | missing^1^ |  | 1 |  |
| Pathological grade | G1/2 | 20 | 30 | 0.659 |
|  | G3 | 16 | 19 |  |
|  | missing^1^ | 6 | 10 |  |
| Smoking status | non-smoker | 6 | 6 | 0.548 |
|  | smoker^2^ | 36 | 53 |  |
|  | missing^1^ |  |  |  |
| Alcohol consumption | non-drinker | 4 | 2 | 0.230 |
|  | drinker^3^ | 38 | 57 |  |
|  | missing^1^ |  |  |  |
| HPV status | non-related^4^ | 30 | 42 | 1.000 |
|  | related^5^ | 10 | 14 |  |
|  | missing^1^ | 2 | 3 |  |

*^1^ data missing; ^2^ former and current smoker; ^3^ former and current drinker; ^4^ viral DNA-negative or DNA-positive but transcript-negative; ^5^ viral DNA- and transcript-positive according to Holzinger et al., 2012 Cancer Res.*

**Supplemental Table S4.** Correlation analysis for FABP5 protein levels and clinical or pathological features of the OPSCC cohort

|  |  | **FABP5^low^** | **FABP5^high^** | **p-value** |
| --- | --- | --- | --- | --- |
| Age [years] | ≤58 | 14 | 34 | 0.667 |
|  | >58 | 17 | 33 |  |
| Gender | male | 21 | 50 | 0.478 |
|  | female | 10 | 17 |  |
| Tumor size | T1/2 | 11 | 28 | 0.658 |
|  | T3/4 | 20 | 38 |  |
|  | missing^1^ |  | 1 |  |
| Lymph node status | N0 | 8 | 11 | 0.411 |
|  | N+ | 23 | 55 |  |
|  | missing^1^ |  | 1 |  |
| Distant metastasis | M0 | 29 | 65 | 0.102 |
|  | M+ | 2 | 0 |  |
|  | missing^1^ |  | 2 |  |
| Clinical stage | I/II | 5 | 6 | 0.321 |
|  | III/IV | 26 | 60 |  |
|  | missing^1^ |  | 1 |  |
| Pathological grade | G1/2 | 14 | 33 | 0.635 |
|  | G3 | 13 | 22 |  |
|  | missing^1^ | 4 | 12 |  |
| Smoking status | non-smoker | 5 | 8 | 0.542 |
|  | smoker^2^ | 26 | 59 |  |
|  | missing^1^ |  |  |  |
| Alcohol consumption | non-drinker | 3 | 3 | 0.377 |
|  | drinker^3^ | 28 | 64 |  |
|  | missing^1^ |  |  |  |
| HPV status | non-related^4^ | 22 | 48 | 0.611 |
|  | related^5^ | 9 | 14 |  |
|  | missing^1^ |  | 5 |  |

*^1^ data missing; ^2^ former and current smoker; ^3^ former and current drinker; ^4^ viral DNA-negative or DNA-positive but transcript-negative; ^5^ viral DNA- and transcript-positive according to Holzinger et al., 2012 Cancer Res.*

**Supplemental Table S5.** Tumor volume (in mm^3^) ± SD in mice (n=4 per group) after injection with either FaDu-mock or FaDu-shALDH1A2 clones.

**
